# Using Machine-Learned Bayesian Belief Networks to Predict Perioperative Risk of Clostridium Difficile Infection Following Colon Surgery

**DOI:** 10.2196/ijmr.2131

**Published:** 2012-09-19

**Authors:** Scott Steele, Anton Bilchik, John Eberhardt, Philip Kalina, Aviram Nissan, Eric Johnson, Itzhak Avital, Alexander Stojadinovic

**Affiliations:** 1 Madigan Army Medical CenterDepartment of SurgeryTacoma, WAUnited States; 2 California Oncology Research Institute (CORI)Los Angeles, CAUnited States; 3 DecisionQ CorporationWashington, DCUnited States; 4 Department of Surgery, Rabin Medical CenterPetah TikvahIsrael; 5 Surgery Branch, National Institutes of HealthBethesda, DCUnited States; 6 Department of Surgery, Division of Surgical Oncology, Walter Reed National Military Medical CenterBethesda, DCUnited States

**Keywords:** Clostridium difficile, Bayesian belief network, pseudomembranous colitis, colectomy, NIS

## Abstract

**Background:**

Clostridium difficile (C-Diff) infection following colorectal resection is an increasing source of morbidity and mortality.

**Objective:**

We sought to determine if machine-learned Bayesian belief networks (ml-BBNs) could preoperatively provide clinicians with postoperative estimates of C-Diff risk.

**Methods:**

We performed a retrospective modeling of the Nationwide Inpatient Sample (NIS) national registry dataset with independent set validation. The NIS registries for 2005 and 2006 were used for initial model training, and the data from 2007 were used for testing and validation. International Classification of Diseases, 9th Revision, Clinical Modification (ICD-9-CM) codes were used to identify subjects undergoing colon resection and postoperative C-Diff development. The ml-BBNs were trained using a stepwise process. Receiver operating characteristic (ROC) curve analysis was conducted and area under the curve (AUC), positive predictive value (PPV), and negative predictive value (NPV) were calculated.

**Results:**

From over 24 million admissions, 170,363 undergoing colon resection met the inclusion criteria. Overall, 1.7% developed postoperative C-Diff. Using the ml-BBN to estimate C-Diff risk, model AUC is 0.75. Using only known a priori features, AUC is 0.74. The model has two configurations: a high sensitivity and a high specificity configuration. Sensitivity, specificity, PPV, and NPV are 81.0%, 50.1%, 2.6%, and 99.4% for high sensitivity and 55.4%, 81.3%, 3.5%, and 99.1% for high specificity. C-Diff has 4 first-degree associates that influence the probability of C-Diff development: weight loss, tumor metastases, inflammation/infections, and disease severity.

**Conclusions:**

Machine-learned BBNs can produce robust estimates of postoperative C-Diff infection, allowing clinicians to identify high-risk patients and potentially implement measures to reduce its incidence or morbidity.

##  Introduction

*Clostridium difficile *(C-Diff) infection has continued to be associated with a steady rise in incidence, increasing over 200% in the United States alone from 2000 to 2005 [1,2]. Stool cultures have demonstrated the underlying gram-positive rod bacterium in approximately 3% of healthy adults, whereas incidence rates are as high as 16% to 35% in hospitalized patients [3,4]. These rates are even higher following prolonged exposure to antibiotics and in patients with underlying cancer or immunosuppression [4]. With this increase, there has been a concomitant rise in one particularly virulent C-Diff strain, 027/B1/North American pulsed-field type 1 (NAP1), that is associated with increased spore formation, higher resistance to fluoroquinolones, up to 23-fold increase in toxin production, and overall worse outcomes [5-7]. This emerging epidemic has not been isolated to the United States, with Canadian reports showing an increase from 0.7 cases per 1000 in 1999-2002 to 14.9 cases per 1000 in 2003-2005 [8]. Additional reports of polymerase chain reaction (PCR) ribotype 027 strains of C-Diff outbreaks across North America and Europe highlight the need for increased vigilance and risk-reducing interventions to prevent its onset [9,10].

Multiple factors contribute to the risk of developing C-Diff colitis. Some of these are well known and easy to monitor, such as antibiotic use and bowel preparations that alter the normal gastrointestinal flora (although controversial) [11-13]. Others are more difficult to pinpoint. The secondary development following elective colonic resection has been shown to be associated with an increased length of stay, higher complication rates, and a nearly 4-fold increase in mortality [14].

Ideally, recognition of patients early in the course of the disease, even with limited data, would allow physicians to initiate treatment in a timely fashion and reduce the likelihood of poor outcomes. A persistent challenge in the treatment of patients who develop C-Diff postoperatively is the absence of a prognostic tool to identify patients who are at high risk of failing standard medical therapy. Identification of patients at an increased risk for C-Diff colitis prior to surgery and implementation of prophylactic strategies could potentially prevent this significant secondary infection altogether. Clinical decision-support systems (CDSSs) have fulfilled an important unmet need to allow for more accurate estimates and predictions where multiple different variables influence disease patterns. CDSSs typically are comprised of a knowledge base that interprets patient-specific information along with a user interface that enables clinicians to interact with the system. The concept is to use specific patient information to make individualized decisions about that patient’s care based on thousands of prior similar scenarios. In other words, “to get the right information needed, to make the right decision, for the right patient, at the right time” [15]. Along with other advances in technology, these have become an essential component of clinical practice in multiple disease processes [16,17].

One such CDSS employs machine-learned Bayesian belief networks (ml-BBNs). These are directed acyclic graphs of conditional probabilities that allow users to understand how different features are conditionally independent of each other. In this study, our objective was to determine if ml-BBNs could preoperatively identify predisposing factors and provide actionable postoperative estimates of C-Diff colitis development following colectomy.

## Methods

### Data Selection and Curation

Data for this study came from the Nationwide Inpatient Sample (NIS), an administrative database provided by the US Department of Health and Human Services and a product of the Healthcare Cost and Utilization Project sponsored by the Agency for Healthcare Research and Quality (AHRQ). This study was performed in accordance with the NIS data user agreement and approval was obtained through a local institutional review board. The NIS is the largest inpatient, all-payer database in the United States, accounting for approximately 8 million hospital admissions each year. It contains information on patient demographics, comorbidities, admission and discharge diagnoses, and multiple outcome measures totaling 220 distinct variables per hospitalization in 2007 alone. Among the data fields are 15 slots for the *International Classification of Diseases, 9* *th* *Revision, Clinical Modification *(ICD-9-CM) diagnosis codes and 15 slots for ICD-9-CM procedure codes. By utilizing a stratified sampling frame and discharge weights, NIS is able to create accurate national estimates from a 20% sample of all nationwide discharges. The states excluded each year per group were different from year to year. The NIS also contains multiple validated severity adjustment measures to estimate patient disease severity used for clinical comparisons. The NIS is described in detail at http://hcup-us.ahrq.gov/nisoverview.jsp (archived at http://www.webcitation.org/6AWhEpjDg).

Patients included in the study were identified within the NIS dataset for the period of 2005-2007 using ICD-9-CM procedure and diagnostic codes. Initial inclusion criteria were patients who underwent colonic resection during hospital admission. All records containing any ICD-9-CM procedure codes beginning with 45.7 (partial excision of large intestine) or 45.8 (total intra-abdominal colectomy) were pulled for analysis because these codes indicate some form of colon resection. A complete list of the corresponding codes, including a summary for each, can be found in Table 1.

**Table 1:** Colon resection ICD-9-CM procedure codes.

Procedure code	Procedure
45.7	Open and other partial excision of large intestine
45.71	Open and other multiple segmental resection of large intestine
45.72	Open and other cecectomy, resection of cecum and terminal ileum
45.73	Open and other right hemicolectomy, ileocolectomy, right radical colectomy
45.74	Open and other resection of transverse colon
45.75	Open and other left hemicolectomy
45.76	Open and other sigmoidectomy
45.79	Other and unspecified partial excision of large intestine
45.8	Total intra-abdominal colectomy, excision of cecum, colon, and sigmoid
45.81	Laparoscopic total intra-abdominal colectomy
45.82	Open total intra-abdominal colectomy
45.83	Other and unspecified total intra-abdominal colectomy

Patients were then identified as having an infection with C-Diff during their admission by searching the NIS NDX-1 secondary diagnosis fields (DX2-DX15) for the ICD-9-CM diagnosis code 008.45 (the code for C-Diff). The primary diagnosis field (DX1) was excluded from this search in order to identify only those hospitalizations in which C-Diff colitis developed following colon resection versus undergoing a colectomy for primary C-Diff colitis [14].

### Definition of Variables

Demographic variables examined included age (years), gender, race, expected payer (ie, Medicare, Medicaid, private insurance, self-pay, or other), type of resection (see Table 1), and median income in the patient’s ZIP code. We also included information on the hospital, such as bed size, control/ownership, region, and teaching status.

The AHRQ comorbidity software, provided by the NIS, was used to examine pre-existing medical conditions. This software assigns variables to identify comorbidities from hospital discharge records using ICD-9-CM diagnosis codes. Comorbidity variables included in our analysis were congestive heart failure (CM_CHF), diabetes (CM_DM), hypertension (CM_HTN), chronic pulmonary disease (CM_CHRNLUNG), renal failure (CM_RENFAIL), peripheral vascular disease (CM_PERIVASC), obesity (CM_OBESE), and malnutrition (CM_WGHTLOSS).

Patient disease severity was accounted for using two validated variables contained within the NIS (provided by the Medstat Disease Staging software, version 5.21): (1) disease staging: principal stage (DS Stage); and (2) disease staging: mortality scale (DS Mtr S). Both variables use several patient-specific parameters present at time of admission to provide a measure of severity for clinical comparison. We used principal stage in our model, which is an assigned numerical value reflective of the level of severity for the principal admitting diagnosis only. For further characterization, we recoded the NIS disease stage variable into 3 basic levels: (1) disease with no complications, (2) disease with local complications, and (3) disease involving multiple sites or systemic complications.

### “InflamAndOtherInfection”

Due to the relationship between antibiotic use and the development of C-Diff colitis, this risk factor was critical for the data analysis. Although the NIS database includes a rich array of information, it does not explicitly identify which antibiotics were administered during the patient’s hospitalization.

To identify risk factors for inflammation and infection, we reviewed the entire list of multilevel clinical classifications software (CCS) categories [18] and used consensus opinion to determine which variables to associate with this category. This culminated in the following infection groupings (and codes): tuberculosis (Tuberculosis), streptococcal septicemia (StreptococcalSepticemia), staphylococcal septicemia (StaphylococcalSepticemia), *Escherichia coli *septicemia (EColiSepticemia), other gram-negative septicemia (OtherGramNegSepticemia), other specified septicemia (OtherSpecSepticemia), unspecified septicemia (UnspecSepticemia), sexually transmitted infection not human immunodeficiency virus or hepatitis (SexTransInfectNotHIVorHep), other bacterial infection (BacterialInfectionOther), any bacterial infection (BacterialInfectionAny), and inflammation/other infection (InflamAndOtherInfection) for CCS codes that did not fit one of the other groups.

### Additional Data Curation

Using an iterative modeling process, the first round of preliminary modeling provided insights on variables and resulted in basic data recoding, such as changing occurrences of ‑8 and ‑88 (undefined variables) to nulls to standardize all missing data within these fields. Additional fields that were conditionally independent of developing C-Diff colitis were censored to simplify the structure of the network and reduce confounders in the model. This was done using structural analysis of the model, such that nodes (ie, variables) that were conditionally independent of C-Diff colitis were removed when they were on the edge of the network.

Clinically, chronic conditions are known when the patient is admitted. To reduce complexity and improve model robustness, the 4 chronic condition variables in NIS (CHRON1, CHRONB1, CHRON2, and CHRONB2*) *were recoded to consolidate their information into 2 variables (CHRONB1mod and CHRONB2mod)*. *The new CHRONB1mod encodes the body system associated with the principal diagnosis, but only if that condition is chronic and the CHRONB2mod variable encodes the body system associated with the second diagnosis only if that condition is chronic.

### Main Outcome Measures

The primary variable in this study was the presence of C-Diff infection following colectomy (ICD-9-CM code 008.45). Again, the NIS diagnosis DX1 was excluded because this would likely indicate admission for primary C-Diff infection and not infection after colon surgery.

### Machine-Learned Bayesian Belief Networks

**Figure 1:**
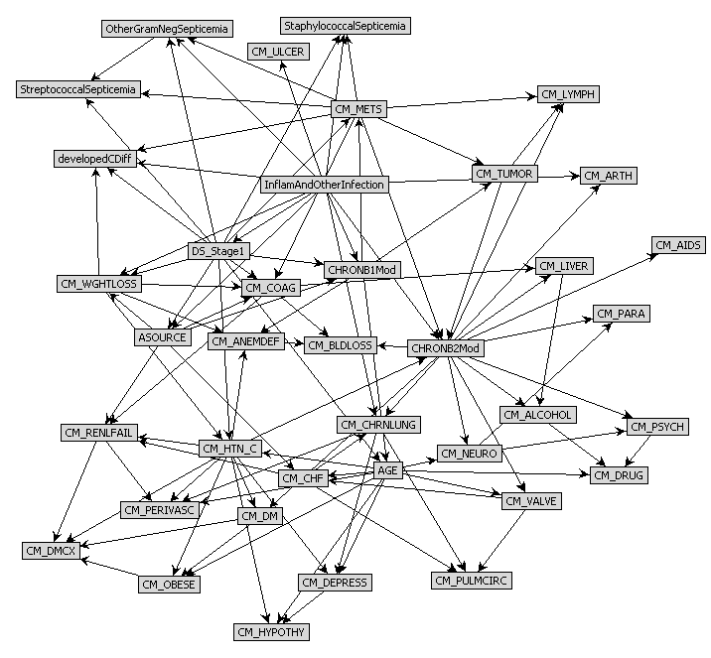
Final pruned focused ml-bbn model.

Machine-learned Bayesian belief networks (ml-BBNs) were trained using commercially available machine-learning algorithms (FasterAnalytics, DecisionQ Corporation, Washington, DC) and a training dataset (NIS 2005 and 2006) to learn network structure and prior probability distributions. The FasterAnalytics software uses heuristic algorithms to allow computers to learn natively from data and discover the most likely structure of conditional dependence between variables in order to specify a BBN. The BBNs are graphs of conditional probabilities that allow users to understand how different features are conditionally independent of each other and to understand how different pieces of information can be used to estimate the likelihood of an outcome. In the present study, this translates to the risk of developing C-Diff infection subsequent to colon resection. We can, for example, identify which data features are first-degree associates of an outcome of interest, or directly conditionally dependent, as indicated by an arc in the BBN graph (Figure 1). Furthermore, because the BBN contains estimates of prior probability distributions as well as joint probability distributions of associated features, by entering observed knowledge into the BBN, it can calculate an estimate of the posterior probability of an event.

More importantly, accurate individual estimates can be made in a multitude of different clinical scenarios, even when all the data points are not known. The object in training ml-BBNs was to focus on postoperative estimates of the risk of developing C-Diff colitis that could be determined preoperatively when given some combination of known demographics, diagnosis and procedure codes, and hospital-level information.

### Training and Validation Data

To develop this model, datasets were obtained from the NIS from the US Department of Health and Human Services for the years 2005-2007. Data were conformed to a common specification as described previously. Data from 2005 and 2006 were used to train the models and data from 2007 were withheld to provide an independent validation set of the model. The objective in using a subsequent year to validate the model (versus k-fold cross-validation or classical statistical analysis) was to provide an independent estimate of model robustness. In essence, it answers the question, “If the model were to be used to assess a new patient population for risk of C-Diff, how would the model perform?” A further benefit of independent set validation is that it tends to produce negatively biased testing results.

To assess model robustness and accuracy, the 2007 NIS independent test set was used to plot receiver operating characteristic (ROC) curves for the final pruned model. ROC curves use the posterior probabilities generated by the model to rank each estimate and compare the trade-off between sensitivity and false positive fraction. Using these curves, we were also able to calculate area under the curve (AUC), a metric of overall classification performance of the model. An AUC < 0.50 is not predictive, whereas an AUC = 1 is perfectly predictive.

As AUCs increase between this range, the model has an overall improved ability to predict outcomes. An AUC between 0.7 and 0.8 is considered “fair” in terms of its ability to predict outcomes. ROC curves can also be used to select the optimal calling threshold by selecting a threshold that optimizes for sensitivity and/or specificity. As such, we ran two threshold cases, a high sensitivity case (70%+) and a high specificity case (70%+), to determine which model was optimized.

### Modeling and Classification

A stepwise model training and feature selection process was used to train the ml-BBNs developed in this study. This consisted of several stages of recursive modeling and data curation, intended to maximize robustness through the selection of an appropriate cohort of data features. This iterative process consisted of (1) preliminary modeling, (2) naïve modeling, (3) global modeling, and (4) focused modeling. At each stage, data features were pruned using a combination of expert knowledge and assessment of model structure, specifically focused on the identification of pathways of conditional dependence with the development of C-Diff infection after colectomy. Additionally, data features were repeatedly assessed to evaluate data quality, resulting in additional curation as needed to further semantically normalize and correct errors within the data. Because clinical registries often have high amounts of unknown or missing data, a passive imputation algorithm was used to impute values for those features in which missing data represented less than 50% of total record count and for which there was no adequate substitute feature.

The first step of the process consisted of a naïve Bayesian model specifying development of C-Diff (developedCDiff) as the dependent variable. Because the NIS dataset is so extensive, the naïve model was used to select features that may be conditionally associated with C-Diff from the overall cohort in order to reduce the complexity of the potential solution set and make the remaining models easier to understand without sacrificing predictive power. Using the naïve model structure, a subset of features was identified to be independently associated with development of C-Diff. This naïve model helped as a guide to decide which variables to retain in the full ml-BBN models. Features suggested by the naïve model, together with the new inflammation/infection features (InflamAndOtherInfection), and the features of the pruned preliminary model were used to define the feature set for a full ml-BBN model.

After selecting a reduced set of features based on naïve analysis, a full ml-BBN was trained in the preliminary modeling step. The objective of this step was to identify confounding effects due to data coding or quality issues, and resulted in additional recoding as discussed previously, such as the combined chronic condition variables and recoding of unknown/missing data to nulls for imputation.

Once additional curation was completed, a set of global models was trained to evaluate individual data features for either pruning or inclusion in the final feature set. Additional variables were pruned from the global model using a combination of expert knowledge and structural evaluation of the classifier. Expert knowledge was applied in 3 areas: (1) to identify those features that were proxies for other variables (identical information under a different name), (2) to identify features that were analogs (not identical to other features, but with highly associated distributions), and (3) to identify features that act as confounders in the model.

Since these three types of features increase the complexity of the model and increase computational time while either reducing or not enhancing robustness, these features were pruned from the final feature list. Additionally, we pruned features that were not included in the ml-BBN using “goodness of fit scoring” from the final feature list. The final list of features was trained as an ml-BBN focused model.

## Results

Our final training cohort consisted of 56,717 colon resection cases in the NIS during 2005 and 54,480 cases in 2006. Rates of C-Diff infection were 1.58% (n = 895) and 1.65% (n = 934) in 2005 and 2006, respectively. Demographics of the training cohort are described in Table 2. The 2007 NIS set consisted of 57,166 cases of colon resection with a rate of C-Diff infection of 1.86% (n = 1064).

**Table 2:** Training population descriptive statistics.

General characteristics		Colon resections without C-Diff (n = 111,368)	Colon resections with C-Diff (n = 1829)	*P*	Total (N = 113,197)
Average length of stay (days)		10.5	23.6	<.001	10.8	
In-hospital mortality, n (%)		5432 (4.9)	336 (18.4)	<.001	5768 (5.1)	
Mean age (years)		62.9	68.3	<.001	63.0	
**Sex, n (%)**						
	Female	59,520 (53.4)	1007 (55.1)	.19	60,527 (53.5)
	Male	51,848 (46.6)	822 (44.9)	.19	52,670 (46.5)
**Disposition (DISPUNIFORM), n (%)**						
	1 = Routine	69,339 (62.3)	409 (22.4)		69,748 (61.6)
	2 or 5 = Other facility	17,787 (16.0)	710 (38.8)		18,497 (16.3)
	6 = Home health	18,616 (16.7)	373 (20.4)		18,989 (16.8)
	20 = Died in hospital	5464 (4.9)	336 (18.4)		5800 (5.1)
	Other	162 (0.1)	1 (0.1)		163 (0.1)
**Disease stage** **^a ^** **(DS_Stage1), n (%)**						
	0	59 (0.1)	0 (0.0)		59 (0.1)
	1	38,973 (35.0)	438 (23.9)		39,411 (34.8)
	2	49,452 (44.4)	647 (35.4)		50,099 (44.3)
	3	22,884 (20.5)	744 (40.7)		23,628 (20.9)
	Mean	1.9	2.2	<.001	1.9
**Admission source, n (%)**						
	1 = Emergency department	34,555 (31.0)	922 (50.4)		35,477 (31.3)
	2 = Another hospital	1968 (1.8)	96 (5.2)		2064 (1.8)
	3 = Another facility including long-term care	936 (0.8)	61 (3.3)		997 (0.9)
	4 = Court/law enforcement	35 (0.0)	0 (0.0)		35 (0.0)
	5 = Routine/birth/other	73,372 (65.9)	738 (40.3)		74,110 (65.5)
	Unknown	502 (0.5)	12 (0.7)		514 (0.5)
**Comorbidities, n (%)**						
	CM_AIDS (Acquired immune deficiency syndrome)	93 (0.1)	3 (0.2)	.24	96 (0.1)
	CM_ALCOHOL (Alcohol abuse)	2163 (1.9)	51 (2.8)	.009	2214 (2.0)
	CM_ANEMDEF (Deficiency anemias)	16,846 (15.1)	348 (19.0)	<.001	17,194 (15.2)
	CM_ARTH (Rheumatoid arthritis/collagen vascular diseases)	2038 (1.8)	47 (2.6)	.02	2085 (1.8)
	CM_BLDLOSS (Chronic blood loss anemia)	5197 (4.7)	97 (5.3)	.20	5294 (4.7)
	CM_CHF (Congestive heart failure	9490 (8.5)	364 (19.9)	<.001	9854 (8.7)
	CM_CHRNLUNG (Chronic pulmonary disease)	18,120 (16.3)	476 (26.0)	<.001	18,596 (16.4)
	CM_COAG (Coagulopathy)	3825 (3.4)	212 (11.6)	<.001	4037 (3.6)
	CM_DEPRESS (Depression)	5482 (4.9)	105 (5.7)	.11	5587 (4.9)
	CM_DM (Diabetes, uncomplicated)	14,787 (13.3)	224 (12.2)	.20	15,011 (13.3)
	CM_DMCX (Diabetes with chronic complications)	1479 (1.3)	50 (2.7)	<.001	1529 (1.4)
	CM_DRUG (Drug abuse)	866 (0.8)	11 (0.6)	.39	877 (0.8)
	CM_HTN_C (Hypertension combine uncomplicated and complicated)	48,181 (43.3)	738 (40.3)	.01	48,919 (43.2)
	CM_HYPOTHY (Hypothyroidism)	8384 (7.5)	116 (6.3)	.06	8500 (7.5)
	CM_LIVER (Liver disease)	1733 (1.6)	39 (2.1)	.048	1772 (1.6)
	CM_LYMPH (Lymphoma)	666 (0.6)	20 (1.1)	.007	686 (0.6)
	CM_LYTES (Fluid and electrolyte disorders)	25,651 (23.0)	881 (48.2)	<.001	26,532 (23.4)
	CM_METS (Metastatic cancer)	16,488 (14.8)	178 (9.7)	<.001	16,666 (14.7)
	CM_NEURO (Other neurological disorders)	3849 (3.5)	125 (6.8)	<.001	3974 (3.5)
	CM_OBESE (Obesity)	5940 (5.3)	53 (2.9)	<.001	5993 (5.3)
	CM_PARA (Paralysis)	1085 (1.0)	37 (2.0)	<.001	1122 (1.0)
	CM_PERIVASC (Peripheral vascular disorders)	3894 (3.5)	103 (5.6)	<.001	3997 (3.5)
	CM_PSYCH (Psychoses)	1841 (1.7)	46 (2.5)	.004	1887 (1.7)
	CM_PULMCIRC (Pulmonary circulation disorders)	684 (0.6)	22 (1.2)	.002	706 (0.6)
	CM_RENLFAIL (Renal failure)	5042 (4.5)	230 (12.6)	<.001	5272 (4.7)
	CM_TUMOR (Solid tumor without metastasis)	2983 (2.7)	62 (3.4)	.06	3045 (2.7)
	CM_ULCER (Peptic ulcer disease excluding bleeding)	61 (0.1)	1 (0.1)	.99	62 (0.1)
	CM_VALVE (Valvular disease)	5444 (4.9)	135 (7.4)	<.001	5579 (4.9)
	CM_WGHTLOSS (Weight loss)	7000 (6.3)	381 (20.8)	<.001	7381 (6.5)
**Infectious variables, n (%)**						
	BacterialInfectionAny	12,693 (11.4)	777 (42.5)	<.001	13,470 (11.9)
	BacterialInfectionOther	4531 (4.1)	139 (7.6)	<.001	4670 (4.1)
	EColiSepticemia	329 (0.3)	12 (0.7)	.005	341 (0.3)
	OtherGramNegSepticemia	472 (0.4)	33 (1.8)	<.001	505 (0.4)
	OtherSpecSepticemia	147 (0.1)	51 (2.8)	<.001	198 (0.2)
	SexTransInfectNotHIVnorHep	18 (0.0)	0 (0.0)	.59	18 (0.0)
	StaphylococcalSepticemia	746 (0.7)	79 (4.3)	<.001	825 (0.7)
	StreptococcalSepticemia	367 (0.3)	30 (1.6)	<.001	397 (0.4)
	Tuberculosis	140 (0.1)	3 (0.2)	.65	143 (0.1)
	UnspecSepticemia	6875 (6.2)	500 (27.3)	<.001	7375 (6.5)
	InflamAndOtherInfection	68,447 (61.5)	1554 (85.0)	<.001	70,001 (61.8)

^a ^Thomson-Reuters/Medstat: stage of principal disease category

Using the 2007 dataset, the final BBN model had an AUC of 0.746, reflecting an acceptable level of predictive capacity. Posterior probability thresholds of 1.2% and 1.5% were used for the high sensitivity and high specificity scenarios, respectively. The high sensitivity case resulted in a sensitivity of 81.0%, specificity of 50.1%, positive predictive value (PPV) of 2.6%, and negative predictive value (NPV) of 99.4%. In contrast, the high specificity analysis had a sensitivity of 55.4%, specificity of 81.3%, PPV of 3.5%, and a NPV of 99.1%.

As a further validation, we determined that some of the comorbidities might be unknown at the time of colon resection. As such, we wanted to estimate robustness and predictive power of the ml-BBN in the absence of features that may not be known at the time of resection.

These features are as follows (with codes): congestive heart failure (CM_CHF), coagulopathy (CM_COAG), hypothyroidism (CM_HYPOTHY), liver disease (CM_LIVER), neurological disorders (CM_NEURO), paralysis (CM_PARA), peripheral vascular disorders (CM_PERIVASC), pulmonary circulation disorders (CM_PULMCIRC), kidney failure (CM_RENLFAIL), peptic ulcer (CM_ULCER), inflammation and other infections (InflamAndOtherInfection), other gram-negative septicemia (OtherGramNegSepticemia), staphylococcal septicemia (StaphylococcalSepticemia), and streptococcal septicemia (StreptococcalSepticemia).

Excluding these potentially unknown or *ex post facto *features, we re-estimated the posterior probability of C-Diff colitis development for each case in our independent validation set and recalculated our validation results. The resulting AUC was 0.743—approximately the same as for the entire set with all variables included.

While using the same posterior probability thresholds, the high sensitivity case had a sensitivity of 77.6%, specificity of 52.0%, PPV of 3.7%, and NPV of 99.1%. The high specificity scenario resulted in a sensitivity of 55.9%, a specificity of 78.9%, a PPV of 6.2%, and NPV of 98.9%. In each situation, this is approximately the same as the full dataset. The similar results are likely due to the highly recursive nature of the model structure.

The final focused ml-BBN structure is described in Figure 1, which represents the conditional independence between associate variables. By reading the structure of the network, we can observe that our outcome of “developed C-Diff” (developedCDiff) has 4 first-degree associates: (1) comorbid metastatic cancer (CM_METS), (2) presence of other, non-C-Diff infections (InflamAndOtherInfection), (3) disease staging (DS_Stage1), and (4) patient weight loss (CM_WGHTLOSS). By incorporating the presence or absence of each of these variables, we were able to develop posterior estimates of the probability of C-Diff infection (Table 3).

**Table 3:** Estimates of *Clostridium difficile *(C-Diff) infection based on presence of risk factors.

Case frequency (%)	Drivers	Target				
	Metastatic tumor	Weight loss	Disease stage	Inflammation/ other infection	Developed C-Diff (%)	
					No	Yes
1.0	No	Yes	Systemic complications	Yes	88.6	11.4
0.0	Yes	Yes	Local complications	No	90.0	10.0
0.1	No	Yes	Systemic complications	No	91.1	8.9
0.0	Yes	Yes	No complications	No	92.3	7.7
0.0	Yes	Yes	No complications	Yes	92.5	7.5
5.1	No	No	Systemic complications	Yes	93.0	7.0
1.0	No	Yes	No complications	Yes	93.7	6.3
2.4	No	Yes	Local complications	Yes	96.3	3.7
0.9	Yes	Yes	Systemic complications	Yes	96.9	3.1
0.2	Yes	No	No complications	No	97.1	2.9
1.1	No	No	Systemic complications	No	97.1	2.9
0.4	Yes	Yes	Systemic complications	No	97.4	2.6
0.0	Yes	Yes	Local complications	Yes	97.7	2.3
0.2	No	Yes	No complications	No	97.8	2.2
0.3	Yes	No	No complications	Yes	97.9	2.1
0.4	Yes	No	Local complications	Yes	98.0	2.0
0.5	No	Yes	Local complications	No	98.3	1.7
24.1	No	No	Local complications	Yes	98.5	1.5
5.3	Yes	No	Systemic complications	Yes	98.5	1.5
21.4	No	No	No complications	Yes	98.8	1.2
0.2	Yes	No	Local complications	No	99.3	0.7
16.6	No	No	Local complications	No	99.4	0.6
11.8	No	No	No complications	No	99.5	0.5
7.1	Yes	No	Systemic complications	No	99.5	0.5

In addition to first-degree associates, the variable developedCDiff, through its first-degree associates also has second-degree associates that can be used to estimate the first-degree associates in patients when there is an absence of information about the first-degree variables (ie, unknown if patient has cancer, weight loss, infection, or staging). With BBN modeling, the user can derive a posterior estimate for the likelihood of C-Diff even with incomplete information. These 15 second-degree associates are as follows (with codes): (1) comorbid lymphoma (CM_LYMPH), (2) comorbid tumor without metastasis (CM_TUMOR), (3) comorbid chronic disease in diagnosis 1 (CHRONB1mod), (4) comorbid chronic disease in diagnosis 2 (CHRONB2mod), (5) comorbid coagulopathy (CM_COAG), (6) comorbid coagulopathy (CM_COAG), (7) streptococcal septicemia (StreptococcalSepticemia), (8) other gram-negative septicemia (OtherGramNegSepticemia), (9) staphylococcal septicemia (StaphylococcalSepticemia), (10) comorbid peptic ulcer (CM_ULCER), (11) comorbid chronic pulmonary disease (CM_CHRNLUNG), (12) admission source (ASOURCE), (13) age (AGE), (14) comorbid hypertension (CM_HTN_C), and (15) comorbid anemia (CM_ANEMDEF) (see Figure 1).

## Discussion

Four first-degree associates that influence the probability of C-Diff development were identified: weight loss, tumor metastases, inflammation/infections, and disease severity. Furthermore, ml-BBNs can produce robust estimates of postoperative C-Diff infection.

The incidence of C-Diff colitis is steadily rising, with most institutions citing rates among all hospitalized patients approaching 1% [19] and as high as 10% in general medical ward patients hospitalized for at least 2 days.

The combination of recent hospitalizations and frequent antibiotic use has led to a near epidemic of chronic carrier states in long-term care facilities. The impact on the health care system is also significant, not only in terms of increased morbidity, but also in terms of escalating costs due to the requirement for patient isolation, personnel protective equipment, and overall care [20]. Although the majority of these patients remain as asymptomatic carriers or only experience mild diarrhea, more fulminant disease may ensue [21]. Yet, C-Diff colitis can also present following elective colonic resection for various disease states ranging from diverticulitis and cancer to inflammatory bowel disease. Among our select cohort of patients undergoing colonic resection, we found a secondary rate of C-Diff colitis of 1.86% for 2007. This is consistent with a slow rise in the years preceding our study, in which the estimated incidence was 14.9 cases per 1000 postoperative hospitalized patients between 2003 and 2005 [8]. Compounding the impact of this recent surge is the accompanying increase in disease recurrence (particularly with the NAP1/B1/027 strain), refractory infections, and the increased clinical severity of cases, with particularly high treatment-related mortality for severe, complicated C-Diff colitis [22]. Previous factors associated with higher morbidity and mortality from C-Diff colitis include low serum albumin, intensive care unit admission, older age [23,24], and poor immunologic response to toxins released by the bacteria [25]. Because each of these factors results in higher rates and disease that is more virulent, identifying those patients at risk and preventing its onset is of paramount importance.

Importantly, current classification systems for C-Diff colitis often understage disease severity, and underscore the need for better models [26].

Estimating the risk of disease-specific outcomes can decidedly improve the management of patients undergoing colonic resection. The goal of this study was, therefore, to create predictive models to provide information on how readily available clinical and disease-specific factors can, in a codependent manner, collectively influence postoperative outcomes through preoperative risk assessment. Machine-learned BBNs have previously been demonstrated to be effective in other areas of medicine, such as estimating risk and prognosis of cancer in patients included in various cancer registries [27,28]. Furthermore, ml-BBNs have the added advantage of providing more accurate estimates when not all the data are known. Although the AUCs of 0.74 and 0.75 predict a “fair” level of predictive capacity, we calculated both high sensitivity and high specificity scenarios to optimize the model. Given the superb model robustness demonstrated through cross-validation in the present study, along with the high degree of variance that can be derived in terms of estimates *a posteriori, *these models provide the basis for an easily usable, personalized medical CDSS even when confronted with limited data.

Given detailed information, the model can also be used as an individualized patient-specific calculator. Table 3 illustrates one mode of using the trained and validated ml-BBN. It uses the 4 first-degree associates—comorbid metastatic cancer (CM_METS), presence of other, non-C-Diff infections (InflamAndOtherInfection), disease staging (DS_Stage1), and patient weight loss (CM_WGHTLOSS)—to estimate the posterior probability of C-Diff given knowledge of these 4 factors. This table represents all possible cases (total 24) within the first-degree associates and their related estimated frequency and posterior probability of C-Diff.

Those cases that exceed the 1.5% threshold (the high specificity threshold) represent an estimated 13.5% of cases, whereas the below-threshold cases represent 86.5% of cases. The value of Table 3 lies in its ability to illustrate how the model can be used to develop estimates of outcome when individual factors are considered collectively. Thus, although weight loss, metastatic cancer, and complications have individual contributions to the likelihood of developing C-Diff colitis, they also have a specific influence on probability when acting together. These estimates are derived from the observed rate of outcome within each subpopulation. In the context of this analysis, variables such as weight loss and systemic complications occur fairly frequently (1 per 100 patients), whereas a case with metastatic disease, weight loss, and local complications is extremely rare (only a handful of patients in our training set). This partly explains our low PPV. Yet, with relatively infrequent incidence in the bigger picture, it is ideal to have a higher NPV, as demonstrated with our model. When expanded to a Web-based application, several other variables (included in Figure 1) could be present in “drop-down” menus in which the provider could place known values, individualizing the patient-specific estimate of disease even further.

A unique aspect of our study is that it evaluates the incidence of C-Diff colitis development following resection for other primary diagnoses rather than focus on surgical therapy of C-Diff colitis itself. This is of particular relevance at a time when the rate of moderate to severe C-Diff colitis is an area of active study covering aspects from vancomycin enemas and fecal transplants, to diverting ileostomy with colonic lavage, or total abdominal colectomy and end ileostomy [29,30].

More pertinent, we were able to identify factors that are often known prior to surgery which increase the risk of the development of C-Diff colitis. These can serve as focal points for intervention such as improving nutrition (for weight loss), treating infection, and optimizing management of systemic disease. With these central efforts, pathways can be implemented to attempt to prevent the onset of C-Diff colitis altogether. Expanding this to an online CDSS will give physicians 24-hour access to input all known data including all the variables (ie, both first- and second-degree associates) to estimate the probability of C-Diff infection following surgery. Decisions could then be made whether to pursue surgery or direct further care prior to surgery. Even beyond the morbidity and mortality, C-Diff infection during hospitalization results in a US $77,000 additional cost per admission, and increases the length of stay by 16 days [31].

We acknowledge some limitations to our study. As in any registry study, there are many issues with data consistency and completeness, as discussed in the Methods section, that required clinical judgment applied to data preparation for analysis. The study team attempted to address these shortcomings through a combination of data curation and censoring, but ultimately these issues cannot be perfectly resolved and we had to rely on the use of ml-BBN independent set validation to assess the impact of database inconsistencies on model accuracy. Also, although the NIS provides a large sample size, it lacks details specific to patients’ hospital courses, including specific antibiotic use, status of chronic carrier states, and degree/severity of comorbid conditions, that could help draw definitive conclusions regarding our endpoints. Finally, the retrospective nature of this analysis likely introduces bias that would not be present in a prospective study.

Despite these limitations, this study does provide useful models that can be easily and readily used to derive case-specific estimates of the development of C-Diff infection for use in identification of high-risk patients and adjusting treatment planning to minimize the onset of C-Diff postoperatively.

### Conclusion

In a large cohort of patients undergoing colonic resection, we have found secondary development of C-Diff colitis to be associated with significant morbidity and mortality. Machine-learned BBN can be used to create robust classifiers capable of estimating the probability of C-Diff colitis preoperatively in patients undergoing colectomy. By identifying high- and low-risk cohorts, physicians can be more aware of patients at additional risk and implement strategies to minimize the probability of secondary C-Diff infection.

## Abbreviations

AHRQ: Agency for Healthcare Research and Quality
AUC: area under the curve
BBN: Bayesian belief network
CCS: clinical classifications software
C-Diff: Clostridium difficile
CDSS: clinical decision-support system
ml-BBN: machine-learned Bayesian belief network
NIS: National Inpatient Sample
NPV: negative predictive value
PPV: positive predictive value
ROC: receiver operating characteristic
